# Study of Polyvinyl Alcohol Hydrogels Applying Physical-Mechanical Methods and Dynamic Models of Photoacoustic Signals

**DOI:** 10.3390/gels9090727

**Published:** 2023-09-07

**Authors:** Roberto G. Ramírez-Chavarría, Argelia Pérez-Pacheco, Emiliano Terán, Rosa M. Quispe-Siccha

**Affiliations:** 1 Engineering Institute, National Autonomous University of Mexico, Mexico City 04510, Mexico; rramirezc@iingen.unam.mx; 2 Research and Technological Development Unit, Research Department, General Hospital of Mexico “Dr. Eduardo Liceaga”, Mexico City 06726, Mexico; argeliapp@ciencias.unam.mx; 3 Faculty of Physical-Mathematical Sciences, Autonomous University of Sinaloa, Culiacan, Sinaloa 80040, Mexico; eteran@uas.edu.mx

**Keywords:** polyvinyl alcohol hydrogels, photoacoustic response signals, state-space model, physical-mechanical properties

## Abstract

This study aims to analyze the physical-mechanical properties and dynamic models of tissue-simulating hydrogels, specifically the photoacoustic (PA) response signals, by varying the concentrations of polyvinyl alcohol (PVA) and molecular weight (MW). A state-space model (SSM) is proposed to study the PVA hydrogels to retrieve the PA-related signal’s damping ratio and natural frequency. Nine box-shaped PVA hydrogels containing saline solution were used, with five concentrations of PVA (7, 9, 12, 15, 20%) for MW1 and four for MW2. The results indicated that the concentration of PVA and MW played an important role in the PA wave’s amplitude, arrival time, and speed of sound over the hydrogels. The SSM parameters showed that increasing PVA and MW concentrations improved the hydrogels’ ability to absorb and transfer energy under the PA effect. These parameters were also found to be correlated with density and modulus of elasticity. Additionally, the concentrations of PVA and MW affected the absorption and optical scattering coefficients. The physical-mechanical properties, including porosity, density, and modulus of elasticity, improved as the concentration of PVA and MW increased. The ultimate goal of this study is to develop hydrogels as phantoms that can be used for tissue simulation and imaging.

## 1. Introduction

Over the past few years, tissue-simulating polyvinyl alcohol (PVA) hydrogels have shown their utility in various functions for biomedical optical and mechanical technologies [1,2,3,4]. The focus has been on developing hydrogels with optomechanical properties similar to those of tissue and using non-ionizing energy to provide a safer modality than X-ray images [5,6]. Regarding the studies of the mechanical properties of the hydrogels, the density, the speed of sound, the Poisson radius, and Young’s modulus have been investigated, changing the number of freezing cycles [7,8]. Furthermore, experiments have been conducted on hydrogels that simulate various tissues, including breasts, to study their absorption and optical scattering coefficients under varying freezing cycles [5,9,10]. In addition, researchers have focused on photoacoustic (PA) wave propagation in hydrogels and biological environments using non-ionizing energy techniques, such as PA tomography. This technique combines photonics with ultrasound and focuses on obtaining images of PVA hydrogels with impurities and biological tissue [10,11,12]. PA tomography works by irradiating the tissue with low-energy laser pulses, the tissue absorbs the laser light and results in thermoelastic expansion, which generates broadband acoustic waves that emanate from the optical absorption volume; the amplitude of this acoustic perturbation is proportional to both local fluence and absorption [13]. Conventional ultrasound transducers can detect these acoustic waves to obtain physical parameters that characterize the sample through dynamic analysis of the PA signals [14] or an image related to the local optical absorption of the tissue [15].

Currently, there is an increasing trend to deploying attractive and robust methods for processing PA signals analysis in an automated way [16]. For instance, the time-domain analysis is commonly performed by seeking the signal amplitude, the envelope, and the time of arrival, which are related to the size, concentration, and distribution of light absorbers in tissue [14]. In contrast, frequency-domain methods analyze the signal power spectrum to relate its bandwidth, central frequency, and magnitude with the size of optical absorbers [17]. Machine learning or data-driven methods have become powerful tools to process PA data, mainly devoted to imaging [18]. However, more information is needed on data-driven methods for processing PA signals to retrieve automated and quantitative results using the raw signals [19,20]. Interestingly, as shown by [20], the PA signal can be considered the impulse response of a dynamical system. Nonetheless, this situation needs to be considered to analyze PA signals, and its applications are limited despite its powerfulness. Therefore, the hypothesis is that a PA signal could be thoroughly described using a dynamical systems approach coupled with data-driven methods [21,22,23].

This study aims to understand how the dynamic model of PA signals and the optomechanical properties of hydrogels vary with two molecular weights (MWs) and different concentrations of PVA. The study analyzed the peak-to-peak amplitude, arrival time, and speed of sound of the PA signals. The physical parameters and state-space models (SSM) were estimated from these signals to determine the damping ratio (absorption capacity), natural frequency (energy transfer through the sample), density, and modulus of elasticity. The optical absorption $\left(\mu_{a}\right)$ and scattering $\left(\mu_{s}\right)$ coefficients were determined using an integrating sphere. Apart from the SSM, the density and the modulus of elasticity were calculated using physical variables and a tensile device. Finally, the ImageJ program and MATLAB were used to calculate the porosity distribution of the microscopic images.

### Dynamical Modeling of Photoacoustic Signals

According to the PA theory, the propagation of acoustic pressure $p(\vec{r},t)$ at position $\vec{r}$ and time *t* is governed by the wave equation:

(1) $$\left(\nabla^{2}-\frac{1}{c^{2}}\frac{\partial^{2}}{\partial t^{2}}\right)p\left(\vec{r},t\right)=-\frac{\beta }{C_{P}}\frac{\partial H(\vec{r},t)}{\partial t},$$

where c is the speed of sound, β is the coefficient of thermal expansion, $C_{p}$ is the specific heat capacity at constant pressure, and $H(\vec{r},t)$ is the thermal energy distributed within the heated region during radiation per unit time. By considering that thermal and stress confinement conditions were met [24] and following a point-source model [25], the generated PA wave at the surface ($\vec{r}=0)$ follows that:

(2) $$\frac{\partial^{2}}{\partial t^{2}}p\left(t\right)+\alpha^{2}\frac{\xi +\frac{4}{3}\eta }{\;\rho \;}\frac{\partial }{\partial t}p\left(t\right)+a^{2}c^{2}p\left(t\right)=\Gamma \frac{\partial H(t)}{\partial t},$$

where α is a propagation constant, ρ is the density, ξ is the bulk viscosity, η is the shear viscosity, and Γ is the Gruneisen parameter.

Interestingly, Equation (2) can be rewritten in terms of a linear-time-invariant (LTI) second-order mechanical system in the following form:

(3) $$\frac{\partial^{2}}{\partial t^{2}}p\left(t\right)+\frac{b}{m}\frac{\partial }{\partial t}p\left(t\right)+\frac{k}{m}\;p\left(t\right)=\frac{u\left(t\right)}{m},$$

where b is the damping coefficient, m is the mass, k is the spring constant, and $u\left(t\right)$ is an external force related to the optical illumination. By comparing Equations (2) and (3), it follows that the natural frequency is $\omega_{n}^{2}=k/m=a^{2}c^{2}$, and the damping ratio is $\zeta =\frac{b}{2m}=\left(\alpha^{2}\xi +\frac{4}{3}\eta \right)/\rho$. Therefore, for PA signals, it is assumed that the system’s response is underdamped, i.e., ζ<1. With the above procedure, we cast the PA problem into a simple dynamical system. Among the wide variety of dynamical model structures, SSM is flexible enough to describe LTI systems at high confidence levels; however, it preserves moderate complexity [26]. Thereby, the model for the PA signal in Equation (3) can be written in SSM form, given by the following equations:

(4) $$\dot{x}\left(t\right)=Ax\left(t\right)+Bu\left(t\right)$$

(5) $$y\left(t\right)=Cx\left(t\right)$$

where $x\left(t\right)\in \;R^{n\times 1}$ is the state vector, $y\left(t\right)$ $\in \;R^{N\times 1}$ is the output sequence, $A\in \;R^{n\times n}$ is the input matrix $B\in \;R^{n\times 1}$ and the output matrix $C\in \;R^{1\times n}$. Herein, the goal is to estimate the SSM matrices using a measured PA signal. For this purpose, system identification provides the tools to estimate the SSM and its parameters from measurements [27]. Following this rationale, by estimating the system-related matrices that produce the output sequence $y\left(t\right)$, it is possible to approximate the measured PA signal $y_{PA}\left(t\right)$. Particularly, to estimate the SSM given in Equations (4) and (5), we used the numerical algorithm for the subspace state-space system identification (N4SID) method to estimate the set of matrices {A,B,C}. A detailed description of the N4SID is given in Section 2.3.

This research aims to provide researchers and clinicians with the tools for constructing hydrogels with varying densities and porosities by adjusting the concentration and molecular weight (MW) to mimic the macro and microenvironment of tissues and enable imaging development. A PVA-based photoacoustic hydrogel was developed using light absorption and speed of sound, optimizing concentration and molecular weight to obtain characteristic optical properties of tissue. The hydrogels were characterized by microscopic imaging methods, mechanical properties, and optical parameters to validate versatility in contrast with a dynamic model.

## 2. Results and Discussion

### 2.1. Photoacoustic Response Signals

**Figure 1:**
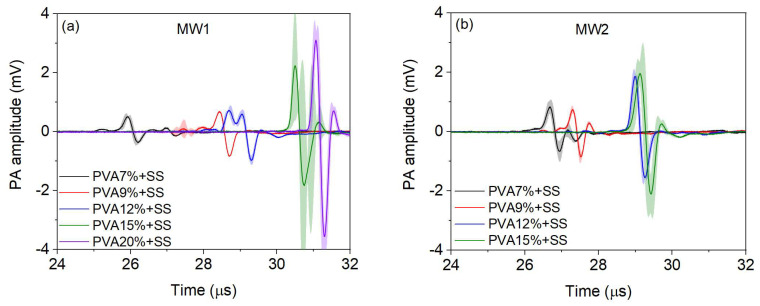
Signals of the PA response of the SS-containing hydrogels as a transmission medium for (**a**) Molecular weight one (MW1) of five PVA concentrations and (**b**) MW2 of four PVA concentrations. Each signal is an average of three measurements.

Figure 1 shows the PA signals of the SS-containing hydrogels as a transmission medium for two MWs and different concentrations of PVA. As can be seen, the amplitude of the signals increases and shifts to longer times when the PVA concentration is higher. When the concentration is higher, the structural stability of the hydrogels improves due to the increase in the number and size of the crystalline regions. At higher MW, the porosity distribution improves due to increased polymer chain length. For example, the arrival time of the 7% and 9% hydrogels of molecular weight two (MW2) are close, and for the 12% and 15% hydrogels, the arrival time becomes very similar. The stability can also be observed in the standard deviation of the amplitude of the signals, and this will depend on the region of the hydrogel wall that the optoacoustic wave passes through until the sensor detects it.

When the PVA concentration increases, the hydrogel diameter expands, and there is a noticeable change in the time-of-arrival sensitivity of PA signals, as seen in Figure 1 and Table 1. Moreover, this time is related to the speed of sound; therefore, it also experiences a slight variation when changing the concentration of PVA, see Table 1. The amplitude of the PA signal is directly related to absorption. Therefore, an increase in PVA concentration results in a higher amplitude of the PA signal, as observed in Table 1.

The laser light passed through the different layers of the hydrogel depending on the PVA concentration and MWs. For higher concentrations, the source absorption was more significant in the first layers of the hydrogel wall due to the larger pore size in those areas than at different depths since there is greater lattice cross-linking at grander depths. However, the intensity of the PA wave did not decrease as it traveled from the absorbing source to the sensor. This was due to the fact that a propagation medium, saline solution (SS), minimized sound dispersion, and the hydrogel structure was more stable at higher concentrations in MWs. It is important to note that the arrival time of the PA signal is later due to the expansion of the hydrogel caused by the increase in the PVA concentration.

Our results are supported by studies showing consistent values for the speed of sound [7,28] and PA amplitude [29]. However, it is important to note that while other researchers changed freezing cycles to determine the speed of sound and PA amplitude, we varied the concentration of PVA.

### 2.2. Analysis of the Dynamical Model

The N4SID algorithm was applied to the measured signals to provide a dynamic model of the samples under study. According to the algorithm presented in Section 4.3, the model order is determined by the SVD of the block Hankel matrix. The N4SID algorithm retrieved a significant magnitude for the measured PA signals for two singular values [30]. The PA signals are fully described by two states of the SSM in Equations (4) and (5), which leads to a model-order $\hat{n}=2$, for both MW. This makes sense since, according to the subspace identification, the magnitude of the singular values of the data monotonically decreases to zero after the second component. Moreover, this behavior only allows one to capture helpful information about the system and neglects the small singular value related to measurement artifacts and noise.

**Figure 2:**
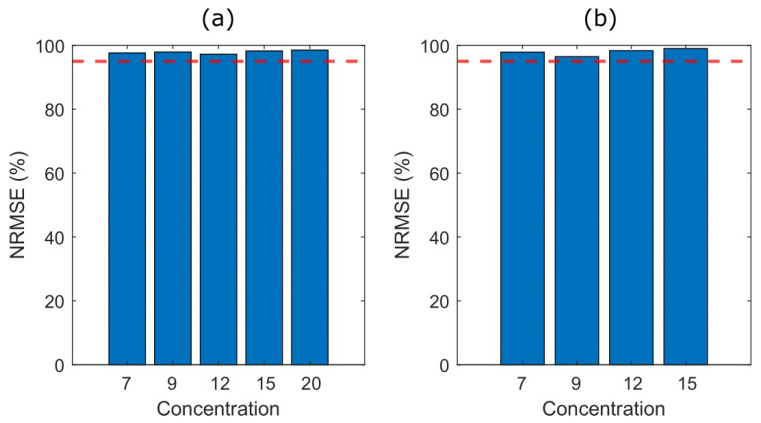
Bar plots of the normalized mean square error (NRMSE) to assess the accuracy of the estimated SSM for (**a**) MW1 of five PVA concentrations and (**b**) MW2 of four PVA concentrations. The horizontal red dashed line highlights the 95% NRMSE.

In order to evaluate the performance of the estimated SSM, the normalized root mean square error (NRMSE) was computed as follows:

(6) $$NRMSE=\left(1-\frac{\left\|y_{PA}\left(t\right)-\right.\left.y\left(t\right)\right\|}{\left\|y_{PA}\left(t\right)-\right.\left.{\bar{y}}_{PA}\left(t\right)\right\|\;}\right)\cdot 100,$$

where $y_{PA}(t)$ is the measured signal, y(t) is the output of the SSM, and ${\bar{y}}_{PA}(t)$ its mean value. Figure 2 depicts the bar plots of the mean NRMSE for the two MWs to assess the similarity between the measured signal and the output of the SSM. In all cases, the estimation accuracy is higher than 95% (horizontal dashed line), thus indicating that a second-order SSM accurately approximates the PA signal for the PVA samples.

As the core of the SSM is the ability to capture information of a dynamic system in a structured form, it is also essential to analyze the parameters of the SSM to provide a physical meaning. Let us recall the SSM in Equations (4) and (5) and that the N4SID algorithm $\hat{n}=2$ estimates the model order. Interestingly, this latter coincides with the order of the model in Equation (3) describing the PA effect. Thus, the PA model in Equation (3) can be explained by an SSM model with eigenvalues of the system matrix $\hat{A}$, given by $\lambda =-\sigma \pm j\omega_{n}$. Moreover, it is well-known that the elements of $\hat{A}$ are related to the natural frequency $\omega_{n}$ and the damping factor ζ as follows:

(7) $$\omega_{n}=|\ln\left(\lambda \right)|\backslash T_{s},\zeta =-\cos(∠ln(\lambda )),$$

these, in turn, are related to the parameters of Equation (3) as $\omega_{n}^{2}=k/m$ and ζ=b/2m. The estimated parameters encode useful parameters that could characterize the MW and concentration of PVA in the samples.

**Figure 3:**
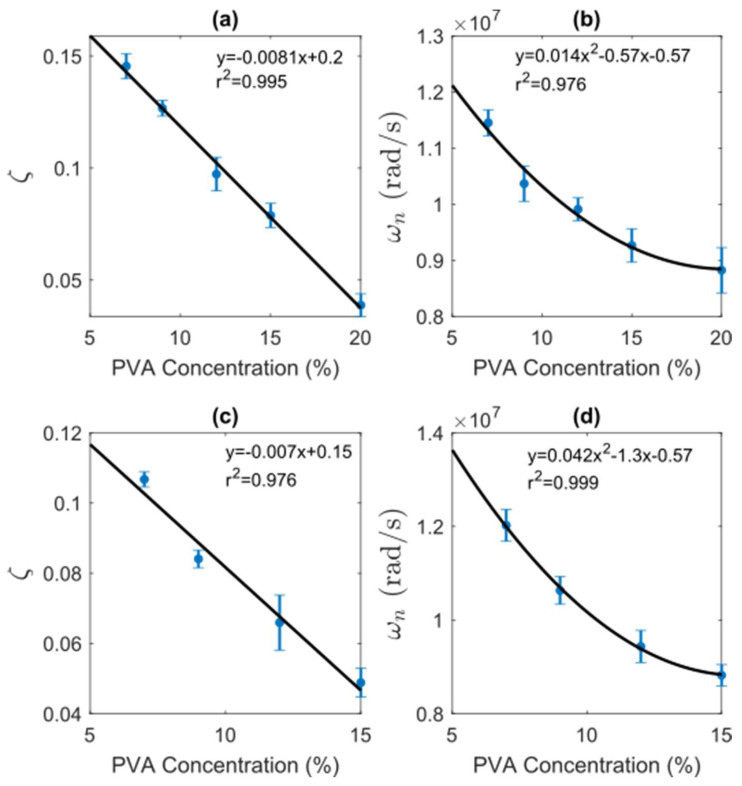
Relationship between state-space model parameters and PVA concentration. (**a**,**c**) show the damping ratio ζ for MW1 and MW2, respectively; (**b**,**d**) depict the natural frequency $\omega_{n}$ for MW1 and MW2.

Figure 3 shows the relationship among the parameters ζ and $\omega_{n}$ retrieved by the SSM and the concentration of PVA for both MWs. Therein, the blue dots represent the sample mean of the estimated parameters, the vertical bars show the relative standard deviation, and the black line is the best fit in the least-squares sense.

In the following, we will discuss the influence of PVA concentrations on the PA signals and their relationship with the SSM parameters.

#### 2.2.1. Damping Ratio

The eigenvalues of the system matrix give the damping ratio. Physically, the damping ratio is dimensionless, defined as how much energy is consumed or how fast the pressure wave dissipates. Therefore, the damping represents the ability to absorb energy after the excited PVA sample. Interestingly, with the proposed SSM estimation, we can directly retrieve this mechanical feature for the measured samples using the PA effect. Figure 3a,c shows the relation between the damping ratio and PVA concentration for the MW1 and MW2, respectively. From there, one can see that ζ exhibits a linear relationship with the concentration of PVA, described by a linear model with above 97% accuracy. This trend could be explained by the fact that the damping ratio reflects the ability of the PVA sample to absorb energy from the pressure produced by the PA effect. Therefore, the PVA concentration could also be related to the elastic behavior of the samples.

#### 2.2.2. Natural Frequency

The natural frequency parameter is retrieved using the imaginary component of the complex conjugate eigenvalue of the model. The natural frequency is related to the so-called resonant frequency, which corresponds to the frequency at which energy can freely transfer back and forth within the sample. Thus, it represents the oscillatory behavior of the PA signal and has a straightforward representation in the frequency domain through a spectrum peak. However, this parameter we show is also encoded by the SSM and is related to the PVA concentration, as shown in Figure 3b,d for MW1 and MW2, respectively. Therein, one can see that the parameter $\omega_{n}$ decreases as the percentage of PVA grows following a second-order degree trend, explained by fitting goodness above 96%. This situation could be attributed to the PA signal’s information loss due to more PVA in the sample. Furthermore, from Figure 3b,d, it is possible to deduce that the MW impacts the bandwidth of the PA signal as it could be related to the density of the sample.

### 2.3. Analysis of Optical Coefficients

Table 2 shows PVA samples’ $\mu_{a}$ and $\mu_{s}$ coefficients and anisotropic parameter (***g***) concerning the PVA concentration. The first column shows the PVA concentration, while the second and third columns display the $\mu_{a}$ and $\mu_{s}$ coefficients. The fourth column shows the reduced scattering coefficient (*µ_s_’)* and the final column outlines the *g* parameter. These values are derived from the optical properties listed in Table 2 in the methodology section.

After analyzing the data from the MW1 samples, it was observed that *µ_a_* increases significantly as the PVA concentration rises from 7% to 15%, reaching a peak value of 2.78 ± 0.02 cm^−1^ at 15% PVA. However, at 20% PVA, the *µ_a_* drops to 0.58 ± 0.01 cm^−1^. Additionally, there is a slight decrease in the *µ_s_* with increased PVA concentration. The *µ_s_* dropped from 54.94 ± 0.55 cm^−1^ at 7% PVA to 52.57 ± 0.42 cm^−1^ at 15% PVA and increased slightly to 53.15 ± 0.42 at 20% PVA. Similarly, *µ_s_’* follows a similar trend and drops from 20.60 ± 0.21 cm^−1^ at 7% PVA to 9.72 ± 0.08 at 15% PVA and increases to 17.54 ± 0.14 cm^−1^ at 20% PVA. Finally, *g* shows significant changes between different concentrations of PVA, with values ranging from 0.62 ± 0.01 to 0.82 ± 0.01, which characterizes the angular distribution of scattered light.

Based on the MW1 sample data, it is clear that higher PVA concentrations lead to greater $\mu_{a}$ and lower *µ_s_* and *µ_s_’* coefficients. Additionally, changes in the *g* indicate that the direction of scattered light depends on PVA concentration. These results reinforce the PA response signals and provide helpful insight into the optical behavior of the samples. This knowledge can help optimize the design of optical biomaterial systems for various biomedical applications.

The *µ_a_* values for the MW2 samples are relatively stable when the PVA concentrations are varied, with a slight decrease observed in the 12% PVA concentration. On the other hand, the *µ_s_* values show a downward trend from 12.86 ± 0.64 to 10.35 cm^−1^ as the PVA concentration increases from 7% to 15%, with the most significant reduction observed in the 9% PVA concentration. Similarly, the *µ_s_’* values show a negative trend from 9.64 ± 0.48 cm^−1^ to 8.07 ± 0.81 cm^−1^ from 7% to 15% PVA, with the minimum value observed in the 9% PVA concentration. The *g* values vary slightly, around 0.25, depending on the PVA concentration. Compared to the MW1 samples, the MW2 samples are somewhat more stable as the *µ_a_* values do not vary much with increasing PVA concentration, even if the *µ_s_* and *µ_s_’* values decrease.

These findings reinforce the results of the PA response signal amplitudes for the MW2 samples since there is better stability. In that case, the 7 and 9% hydrogels showed similar amplitudes, and the 12 and 15% hydrogels showed comparable amplitudes when the PVA concentration was varied, with the most significant increase in amplitude being from the 9% PVA hydrogel to 12%.

The values of this study’s $\mu_{a}$ and optical $\mu_{s}$ coefficients align with findings reported in other studies [5,6]. They determined the optical coefficients by changing the freezing cycles, and we determined them by varying the PVA concentration. The differences in the location of the sample and its internal structures may contribute to variations in the data observed.

Hydrogels’ optical stability depends on their absorption and scattering capacity of near-infrared light, which varies with concentration. Increasing PVA concentration improved absorption capacity for both MWs. MW1 has a lower $\mu_{a}$ and higher $\mu_{s}$ coefficient for 7% and 9% concentrations, while higher concentrations of 12%, 15%, and 20% have higher $\mu_{a}$ and lower $\mu_{s}$ coefficients. MW2 has similar $\mu_{a}$ and $\mu_{s}$ coefficients for all concentrations except for 7% and 9%, with higher $\mu_{a}$ and lower $\mu_{s}$ coefficients than MW1. These hydrogels mimic tissue optical properties and aid in diagnosis and treatment using optical techniques such as PA and integrating sphere.

### 2.4. Analysis of Porosity Distribution

**Figure 4:**
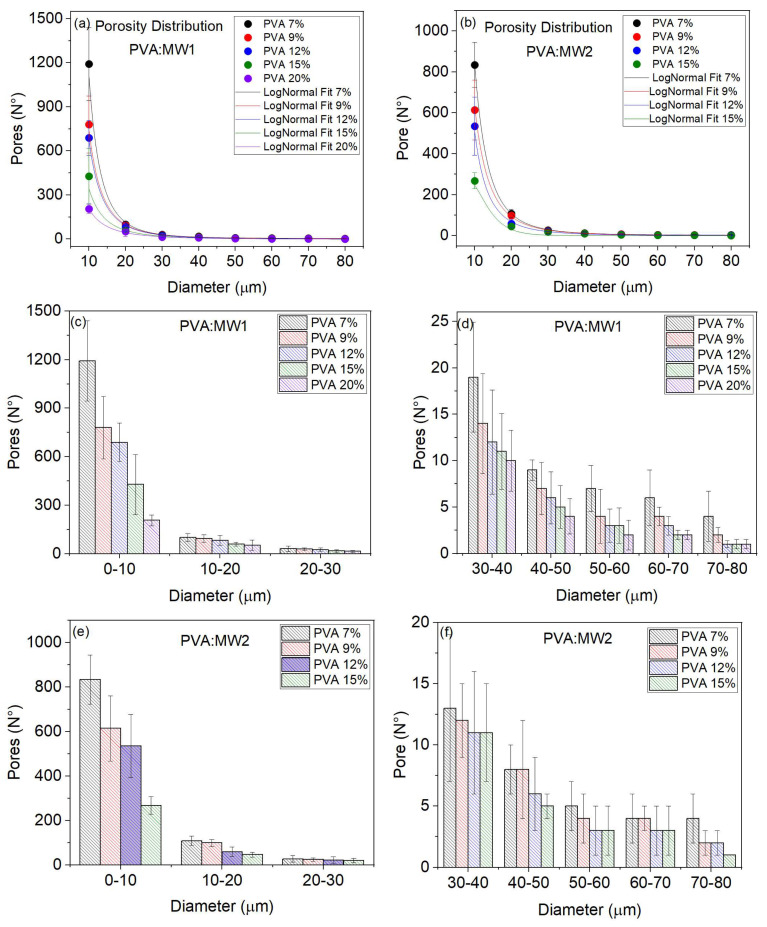
The behavior of the porosity distribution fitted to a LogNormal curve with R^2^ = 0.998 for (**a**) MW1 samples and (**b**) MW2 samples. Additionally, the porosity distribution is displayed for both (**c**) small-diameter groups (0–10 µm, 10–20 µm, and 20–30 µm) and (**d**) large-diameter groups (30–40 µm, 40–50 µm, 50–60 µm, 60–70 µm, and 70–80 µm) for MW1 samples, and for the MW2 samples are (**e**) small-diameter groups and (**f**) large-diameter groups.

This section presents the results of the porosity distribution (number of pores versus diameter) for the MW1 and MW2 samples. The diameter range of the pores identified in the samples for different concentrations of PVA was from 10 to 80 µm. The increase in the concentration of PVA leads to a decrease in the diameter of the pores and, consequently, the number of pores. High MW samples have greater cross-linking due to more polymer chains; therefore, the pore diameter decreases. The porosity distribution for the samples of the two MWs fits a LogNormal curve with a value R^2^ = 0.998, as shown in Figure 4a,b. Each measurement is the average of four samples.

The histograms in Figure 4b,c display the distribution of porosity, categorized into two groups: small diameters ranging from 0–10 µm, 10–20 µm, and 20–30 µm, and larger diameters ranging from 30–40 µm, 40–50 µm, 50–60 µm, 60–70 µm, and 70–80 µm. The number of pores is higher for smaller diameters, notably within the 0–10 um range. As the concentration of PVA increases, the number and diameter of pores decrease. This trend is also observed in the MW2 samples, where increasing PVA concentration leads to fewer pores, as shown in Figure 4e,f. The differences in the measurements may be attributed to the composition of the various sample cuts.

When the samples have high concentrations and MW, their physical-mechanical properties improve and become more viscous. However, this also leads to a higher reduction in permeability. Our findings can assist in selecting the optimal parameters for PVA hydrogels in biopolymer applications.

### 2.5. Analysis of Density and Elasticity Modulus

**Figure 5:**
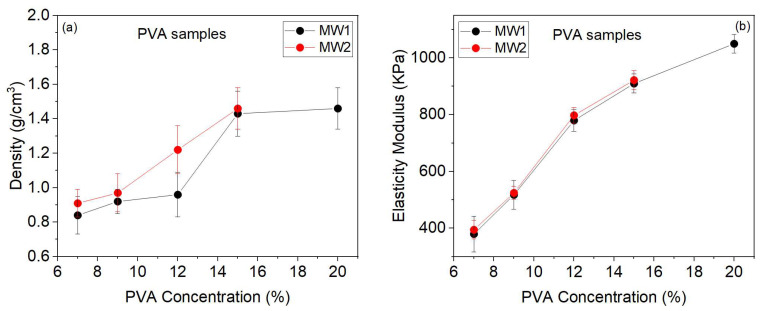
Mechanical properties of the PVA samples with two MWs and different concentrations. The density and elasticity modulus are plotted in (**a**,**b**), respectively.

Figure 5a shows that higher concentrations increase density due to decreased porosity, as previously discussed. The densities of MW2 are slightly higher than those of MW1. In both MWs, the densities of the 9% samples are slightly higher than those of 7%. However, the increase in density is more significant for the 12% and 15% samples compared to the 7% and 9% samples. The increase in density between 15% and 20% of MW1 is very smooth. The standard deviation of the density values is due to the porosity distribution of the four layers cut from the hydrogel wall for each concentration.

The concentration of PVA directly impacts the modulus of elasticity and density. As the concentration of PVA increases, the modulus of elasticity also increases. The modulus of elasticity increases significantly for higher concentrations of PVA, as seen in Figure 5b. It is important to note that measurement variations may occur due to cutting different sections from the hydrogel wall.

Instead of measuring the modulus of elasticity with the number of freezing cycles, we report it based on concentration. Our findings are comparable to previous studies by [7,31]. It is important to note that the sample preparation methods, such as MW, concentration, freezing cycles, freezing time and temperature, and sample dimensions, play an essential role in the results. Our study’s density and modulus of elasticity are consistent with the tissue simulant hydrogels’ results for endoscopic ultrasound images due to using the same manufacturing methodology, as reported in [3].

After analyzing the modulus of elasticity, it was observed that the samples from MW2 are slightly higher than those of MW1. This means that the polymer with higher MW has more chains, resulting in fibers with greater tensile strength.

In this study, the mechanical stability of hydrogels depends on porosity, density, and modulus of elasticity. Low PVA concentrations (7% and 9%) result in greater pore diameter and number, leading to higher swelling and permeability but lower tensile strength. This behavior is similar for both MW1 and MW2. Hydrogels with higher PVA concentrations (12%, 15%, and 20%) have smaller pore diameters, higher density, and tensile strength, making them suitable for tissue simulation and image characterization.

### 2.6. Relationship of Dynamical Model and Mechanical Properties

**Figure 6:**
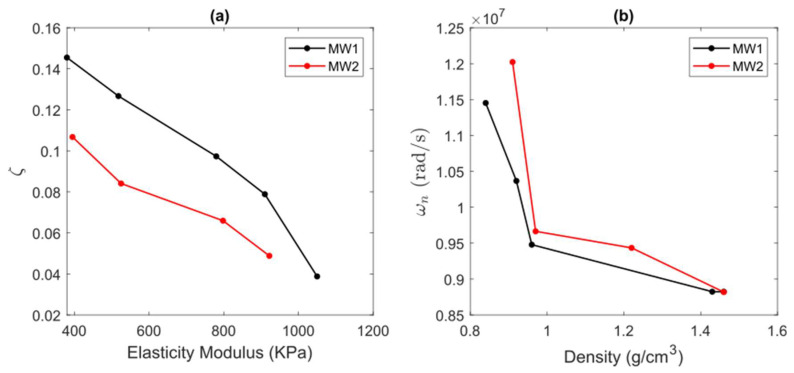
Relationship between state-space model parameters and mechanical properties of PVA samples for two MWs and different concentrations, (**a**) damping ratio versus elasticity modulus, and (**b**) natural frequencyversus density.

For comparison purposes, we investigated the relationship of the elasticity modulus and density with the damping ratio and natural frequency, respectively, as shown in Figure 6. From Figure 6a, one can see that the damping ratio exhibits a quasi-linear inverse relationship with the elasticity modulus as the concentration grows for both MWs. This situation indicates that the concentration of PVA is related to the samples’ elasticity; therefore, the damping ratio could be an effective parameter to characterize it using the PA effect. On the other hand, from Figure 6b, the natural frequency is inversely related to the sample density for different concentrations. In particular, using the natural frequency of the PVA samples, it is possible to infer the density of the samples, as suggested by the PA model in Equation (2).

## 3. Conclusions

This study found that the concentration and MW changes affected the polymeric network’s cross-linking and the hydrogels’ dimensions. For that reason, the arrival time shifted toward longer times with increasing PVA concentration, and the amplitude of the PA signal increased, whereas the latter was due to the superior PVA absorption. A similar behavior was observed for the MW2 samples but with an amplitude slightly more pronounced. Moreover, as the speed of sound is related to arrival time, it displayed variations with increasing PVA concentration. Additionally, a subspace identification (SI) algorithm allowed for the recovery of state-space models from the temporal and frequency profile of the PA signals containing the sample’s biophysical information. There was a similarity between the measured signal and the SSM signal using the NRMSE, which allowed for capturing valuable details about the system and depreciating small noise-related singular values. Based on the damping ratio, it was observed that an increased concentration of PVA resulted in higher energy absorption capacity of the hydrogels under the PA effect for both MWs. Concerning the natural frequency, it was observed that higher PVA concentration led to increased energy transfer in the hydrogels for both MW. Intriguingly, the damping ratio and natural frequency correlated with the modulus of elasticity and density. These properties were observed to increase with higher PVA concentration.

On the other hand, as the concentration of PVA increased, the $\mu_{a}$ coefficients also improved while the $\mu_{s}$ coefficient decreased slightly. Nevertheless, the MW2 samples exhibited minimal changes in their $\mu_{a}$ coefficients and optical $\mu_{s}$, remaining almost steady. These results provide further evidence for the conclusions drawn from the PA method. From the distribution results of porosity, density, and modulus of elasticity, it can be concluded that the physical-mechanical properties of the hydrogels were improved as the concentration of PVA and MW increased. This study aimed to enhance the characterization of hydrogels with varying PVA concentrations and MW through diverse methods. The goal is to create hydrogels as phantoms that can be applied explicitly in tissue simulation and imaging.

## 4. Materials and Methods

### 4.1. PVA Hydrogels

In this study, nine PVA hydrogels shaped as hollow boxes were used. Five PVA boxes were obtained with concentrations of 7%, 9%, 12%, 15%, and 20% for MW1 = 85,000–124,000, and four PVA boxes with concentrations of 7%, 9%, 12%, and 15% for MW2 = 146,000–186,000. Regardless, the 20% PVA hydrogels could not be obtained due to the complexity of its preparation in the gel stage, which resulted in lumps.

This study chose a wide range of PVA concentrations to characterize the hydrogels through physical-mechanical techniques and analyzing PA signals using a dynamic model, in order that they can be applied as tissue simulators (organs and lesions) [3,4,5,6,28,32] and images [10,11,13,15,33].

To obtain the hydrogels with different concentrations, two steps were followed:

*First step: PVA gel*

For the preparation of PVA gel, powder with 99% hydrolysis (Sigma Aldrich, Saint Louis, MO, USA) was used, and to obtain the weight–concentration relationship, the following equation was used:

(8) $$W_{PVA}\left(mg\right)=[\left(PVA\;concentration\;\left(\%\right)-H_{2}O\;volume\;\left(\mathrm{mL}\right)\right]/100\%$$

The gel is obtained from the mixture of 150 mL pure water Milli-Q^®^ (Merck, Darmstadt, Germany) and 10.5 mg of PVA powder corresponding to the concentration of 7% PVA by heating from 20 °C to 85 °C for approximately 90 min. The mixture was stirred with a bar magnet throughout the heating process to ensure a homogeneous solution. Then, this gel is left to rest to cool down and reach room temperature (20 °C) and eliminate air bubbles that may be trapped in the solution. The same procedure was followed for the rest of the concentrations, only changing the weight corresponding to each concentration.

*Second Step: PVA hydrogel*

**Figure 7:**
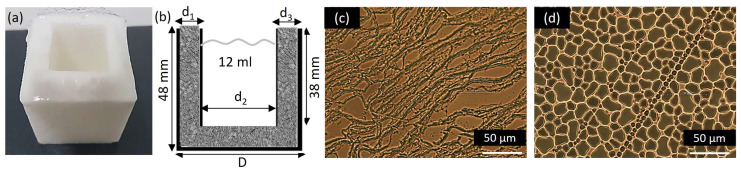
(**a**) PVA hydrogel image (MW1, 12%), (**b**) hydrogel cross-section dimensions (contains 12 mL of saline solution), (**c**) microscopic image of a 10 µm dehydrated film of the PVA hydrogel (scale, 50 µm), and (**d**) the same PVA film as (**c**) with a drop of water (Nikon optical microscope, Tokyo, Japan 40X objective, 10X eyepiece).

The gel obtained in the first step is poured into a mold in the form of a box (coated steel) to undergo the process of freezing (−80 °C) using a freezer (Kaltis GV039M, Shanghai, China) during 1.5 h and thawing to room temperature (20 °C) during 4 h for four cycles. Finally, a PVA hydrogel is obtained, as shown in Figure 7a. To keep the hydrogel for a long time (even years), it is left submerged in pure water.

The hydrogel dimensions are represented in Figure 7b, where d_1_ and d_3_ are the thicknesses of the hydrogel walls, d_2_ is the internal diameter, and D is the diameter. This hydrogel contains 12 mL of SS to propagate the PA wave when laser light irradiates. The hydrogels’ defined dimensions and hollow shape were due to two reasons: the area needed to be greater than the sensor’s surface area to avoid laser light scattering, and the hydrogels needed to accommodate biological substances and tissue, such as mammary tumors, fibroadenomas, microcalcifications, etc.

Regarding microscopic images in Figure 7c, a 10 µm dehydrated film is shown. This was cut from a section of the hydrogel wall, and a drop of water on the dehydrated film is observed in Figure 7d.

By increasing the concentration of PVA in the hydrogels, a slight expansion of their dimensions, d_1_, d_2_, and d_3_, is observed in Table 3. This is a direct result of the greater crosslinking of the hydrogel, which leads to a higher degree of crystallinity and, subsequently, a higher resistance to mechanical stress.

Figure 7c shows a microscopic image (Nikon optical microscope, 40X objective, 10X eyepiece) of a 10 µm thick film that was obtained from the PVA hydrogel wall, and it was dehydrated for 24 h. Figure 1d shows the same film but hydrated with one drop of pure water, where it is observed how the pores are filled with this solution. The PA signals obtained in this study were from the empty hydrogel and with propagation medium, 12 mL of SS or sodium chloride (NaCl), 0.9% (Baxter, Jiutepec, Morelos, Mexico).

### 4.2. Photoacoustic Method

**Figure 8:**
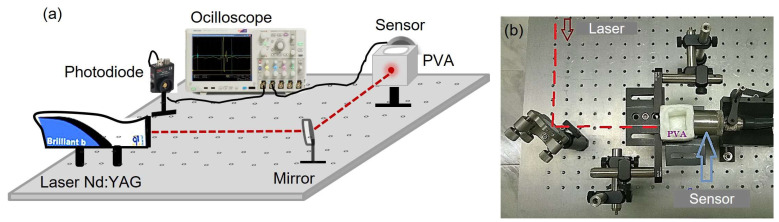
(**a**) Schema of the PA experimental setup to obtain the PA response signal of the hydrogels with two MWs and different PVA concentrations, (**b**) photography of the PA experimental setup.

The PA signals of the PVA hydrogels of two MWs and different concentrations were obtained by the pulsed PA technique, as seen in Figure 2. The experimental setup consists of an Nd: YAG laser (Brilliant B, Quantel, Lannion, Côtes-d’Armor, France) with λ = 1064 nm, τ=6 ns, F = 10 Hz, and fluency = 33 mJ/cm^2^ per pulse. An oscilloscope (DPO 5204B, Tektronix, Beaverton, OR, USA) with a bandwidth of 2 GHz and a sampling rate of 10 Gs/s to record the signals, a 100% reflectance mirror (NB1-K14, Thorlabs, Newton, NJ, USA) to reflect the laser beam, a 2.25 MHz immersion transducer (I-8 series, Olympus, Tokyo, Japan) to detect the ultrasonic signals, a Si photodetector amplified (PDA10A, Thorlabs, Newton, NJ, USA) from 200–1100 nm to detect the laser shot, and finally the PVA hydrogel with SS, which is adjusted laterally by two clamps to try to maintain the same sensor coupling with all hydrogels (Figure 8b).

This technique involves incising laser light on the hydrogel that is coupled directly to the sensor, part of this light is absorbed by the hydrogel, and the other part is scattered. Then, the absorbed part (absorption source) is heated, generating a thermoelastic effect and emitting optoacoustic waves that travel through the SS medium and are detected by an ultrasound sensor and recorded by the oscilloscope. Initially, the hydrogel is empty, and its PA signal is recorded. Then, 12 mL of SS is added, and the signal is recorded again. This process is repeated for each hydrogel three times.

As PVA hydrogels have high water absorption capacity, the coupling with immersion transducers is excellent since these are used to being in contact with water.

#### Photoacoustic Response Signals

**Figure 9:**
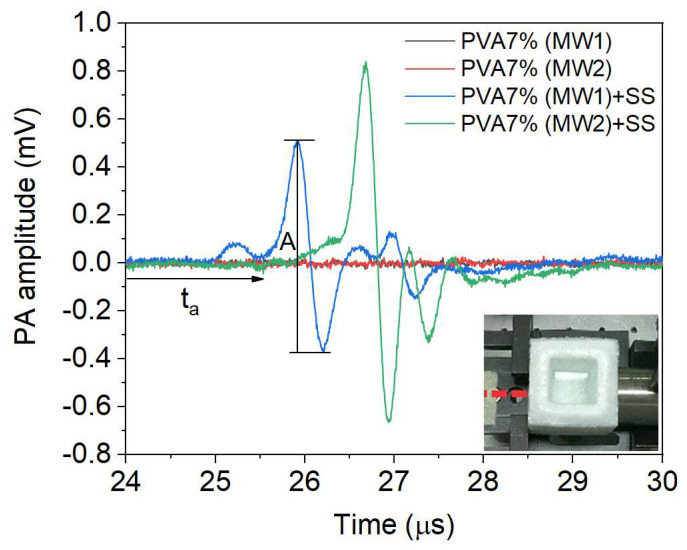
Amplitude versus time of PA signals for MW1 and MW2 of a PVA 7% hydrogel, both empty and with a saline solution medium.

In Figure 9, we can see the PA signals of an empty hydrogel and one containing SS (in this case, a PVA 7% hydrogel for MW1 and MW2). The empty hydrogel does not show a PA response since there is no medium for the acoustic waves generated in the hydrogel wall to propagate toward the sensor. However, it is important to understand that the acoustic wave is generated by the vibrations of the hydrogel structure when it absorbs electromagnetic energy. This structure is a 3D network containing water in its micropores and comprises a crystalline and amorphous phase. Therefore, increased PVA concentration and MW will be noted in the amplitude and delay time of the PA signal.

The image attached to Figure 3 shows that the beam impinges directly on the PVA wall, produces pressure waves, and travels through the medium SS until the sensor detects them.

By analyzing the PA signal, we can determine the arrival time of the pressure wave ${(t}_{a})$, which indicates how long it takes to travel from the source of absorption to the sensor. Peak-to-peak amplitude (*A*) can also be measured, directly related to absorption. Additionally, we can calculate the speed of sound (*c*) using the equation $c=D/t_{a}$, where (*D)* is the hydrogel diameter.

The PA signal amplitude is related to the PVA concentration and arrival time with the hydrogel’s porosity distribution (number and diameter of pores) by varying the concentration.

### 4.3. Dynamical Model Identification

One of the most promising methods to derive data-driven models in an SSM structure is the SI approach [31]. SI aims to estimate the SSM in Equations (4) and (5) using structured measurements of input/output signals and orthogonal projections. Particularly, in this study, we appeal to the numerical algorithm for the N4SID method owing to its numerical stability. The N4SID is an algebraic in nature method working as follows. Let the measured PA signal $y_{PA}\left(t\right)$ be arranged in a block Hankel matrix defined as

(9) $$\left[\frac{Y_{p}}{Y_{f}}\right]=\left[\frac{\begin{array}{ccc} y_{0} & \cdots & y_{j-1}\\ \vdots & \ddots & \vdots \\ y_{i-1} & \cdots & y_{i+j-2} \end{array}}{\begin{array}{ccc} y_{i} & \cdots & y_{i+j-1}\\ \vdots & \ddots & \vdots \\ y_{2i-1} & \cdots & y_{2i+j-2} \end{array}}\right],$$

where $Y_{p}$ and $Y_{f}$ are the matrices of past and future, respectively. Moreover, a shifted version of the Hankel matrix is constructed by moving the first block row of $Y_{f}$ to the bottom of $Y_{p}$, thus leading to:

(10) $$\left[\frac{Y_{p}^{+}}{Y_{f}^{-}}\right]=\left[\frac{\begin{array}{ccc} y_{0} & \cdots & y_{j-1}\\ y_{1} & \cdots & y_{j}\\ \vdots & \ddots & \vdots \\ y_{i} & \cdots & y_{i+j-1} \end{array}}{\begin{array}{ccc} y_{i+1} & \cdots & y_{i+j}\\ \vdots & \ddots & \vdots \\ y_{2i-1} & \cdots & y_{2i+j-2} \end{array}}\right],$$

Similarly, the input signal and the state sequence can be arranged following the notation in Equations (9) and (10). Moreover, a regressor is formed by the combination of $U_{p}$ and $Y_{p}$ as $W_{p}={{[U}_{p}{\;Y}_{p}]}^{T}$, which will be used further to solve the identification problem. Let us define the following:

(11) $$\Gamma ={\left[C\;CA\;CA^{2}\;\cdots \;CA^{i-1}\right]}^{⊤},\Delta =\left[A^{i-1}B\;A^{i-2}B\;\cdots \;B\right],$$

as the extended observability and controllability matrices, respectively. Finally, the impulse response is given by the following Toeplitz matrix:

(12) $$H=\left[\begin{array}{cccc} 0 & 0 & \cdots & 0\\ CB & 0 & \cdots & 0\\ CAB & CB & \cdots & 0\\ \vdots & \vdots & \ddots & 0\\ CA^{k-2}B & CA^{k-3}B & \cdots & 0 \end{array}\right]$$

At this point, two main assumptions should hold for our particular problem: (i) The system governing the PA signal is stable, observable, and controllable, and (ii) the input signal excites the useful modes to be modeled.

The problem here is, given a set of PA signal measurements, to estimate the parameters of the SSM using subspaces of the matrices presented above. For this purpose, the SSM can be rewritten in matrix notation as the following system of linear equations:

(13a) $$Y_{p}=\Gamma_{i}X_{p}+H_{i}U_{p},$$

(13b) $$Y_{f}=\Gamma_{i}X_{f}+H_{i}U_{f},$$

(13c) $$X_{f}=A^{i}X_{p}+\Delta_{i}U_{p},$$

where Equations (13a) and (13b) define past and future outputs as a linear combination of previous states and the extended observability matrix. On the other hand, Equation (13c) relates the future and past states given the system matrix A.

Now, the algorithm aims to estimate the subspaces of the matrix Γ and the state sequence $X_{f}$, which can be obtained from data projections. Let $O_{i}$ be an orthogonal projection defined by $O_{i}=\Gamma_{i}X_{f},$ which can be computed by the projection of the future output onto the subspace of past data in the direction of future input as:

(14) $$O_{i}=Y_{f}{/}_{U_{f}}W_{p},$$

Thereafter, it can be factorized using singular value decomposition (SVD), in order that:

(15) $$O_{i}=U\Sigma V^{⊤}=\left(U_{1}U_{2}\right)\left(\begin{array}{cc} S_{1} & 0\\ 0 & 0 \end{array}\right)\left(\begin{array}{c} V_{1}^{⊤}\\ V_{2}^{⊤} \end{array}\right)=U_{1}S_{1}V_{1}^{⊤},$$

where $S_{1}$ is a diagonal matrix containing the non-zero singular values of the matrix $O_{i}$. Moreover, the required subspaces are computed as:

(16) $$\Gamma_{i}=U_{1}S_{1}^{1/2},$$

which, in turn, is useful to solve the relationship in Equation (14). By construction, it holds that using Equations (14) and (16) is possible to estimate the state sequences given that:

(17) $${\hat{X}}_{i}=\Gamma_{i}^{ϯ}O_{i},\;{\hat{X}}_{i+1}=\Gamma_{i}^{ϯ}O_{i+1},$$

where the subscript ϯ denotes the pseudo-inverse. Once the state sequences are estimated, the following set of equations can be solved:

(18) $$\underset{F}{\underbrace{\left(\begin{array}{c} {\hat{X}}_{i+1}\\ Y_{i} \end{array}\right)}}=\underset{\theta }{\underbrace{\left(\begin{array}{cc} A & B\\ C & 0 \end{array}\right)}}\underset{M}{\underbrace{\left(\begin{array}{c} {\hat{X}}_{i}\\ U_{i} \end{array}\right)}},$$

which is a SSM in matrix notation. Interestingly, Equation (18) has a closed-form solution which can be algebraically computed. Thus, the optimal set of parameters $\theta^{⋆}$, in the least-squares sense, is given by $\theta^{⋆}=FM^{⊤}{\left(MM^{⊤}\right)}^{-1}$, that corresponds to an estimate of the SSM matrices, $\hat{A},\;\hat{B}$, and $\hat{C}$. Thereby, the estimated SSM is related with the impulse response function governing the PA signal [32] and, in this way, one can perform a parametric analysis of PA, given the elements of the system-related matrices.

### 4.4. Optical Coefficients

The optical properties of the hydrogel’s samples were investigated using a setup depicted by Teran et al. [34]. We utilized five different illumination beams to examine the diffuse reflectance and transmittance of these hydrogels. Each beam had a specific purpose: beam 1 provided diffuse illumination for reflection measurements (power detected, *P_d_*); beam 2 offered collimated illumination (power detected, *P_r_*); beam 3 accounted for the sample’s specular reflection; beam 4 aided in transmission measurements with collimated illumination (power detected, *P_t_*); and beam 5 represented the light that passed through the sample without scattering (power detected, *P_coh_*). Switching between these beams was facilitated by removable mirrors.

For this setup, we utilized an integrating sphere (ISP-50-8-R-GT Ocean Optics) with an internal diameter of 13.46 cm and a wall reflection coefficient of *m = 0.98*. This sphere played a vital role in determining the power that the detector received under different illumination conditions. Using mathematical equations based on integrating sphere theory, we estimated this power for each beam and then determined the total reflectance and transmittance of the sample.

To determine the directional-diffuse reflectance, we compared the power reflected in a directional-diffuse illumination setup (beam 2) to the power detected in a diffuse-diffuse illumination setup (beam 1). This reflectance value is represented as:

(19) $$R_{cd}=mP_{r}/P_{d}$$

On the other hand, we calculated the directional-diffuse transmittance (referred to as beam 4) by comparing the power detected in this configuration to the power detected in the diffuse-diffuse configuration:

(20) $$T_{cd}=mP_{t}/P_{d}$$

We calculated the coherence transmittance by measuring both the incident power (*P_o_*) and the directional light transmitted through the sample (*P_coh_*):

(21) $$T_{c}=P_{coh}/P_{o}$$

The relationships between reflectance and transmittance, including both coherent and diffuse measures, are crucial in determining the inherent optical properties of the samples.

Table 4 presents the optical properties of the hydrogel samples as a function of the PVA concentration, as described by Equations (19)–(21). The optical properties under consideration include diffuse-directional reflectance (*R_cd_*), diffuse-directional transmittance (*T_cd_*), and coherence transmittance (*T_c_*)—each sample with a width of 100 µm.

In the case of MW1 samples, it was noticed that the *R_cd_* decreased as the concentration of PVA increased. This decrease was particularly significant at higher PVA concentrations, with the lowest value of 0.14 ± 0.01 observed at 15% PVA. However, at 20% PVA, the *R_cd_* increased again to a value of 0.34 ± 0.01. These findings suggest that the concentration of PVA plays a crucial role in determining the reflectance behavior of the samples. Similarly, the *T_cd_* showed a similar pattern, with a general decrease observed as the concentration of PVA increased. At 7% PVA, the *T_cd_* was 0.47 ± 0.01, which gradually decreased to 0.41 ± 0.01 at 15% PVA. For the highest concentration of 20% PVA, the *T_cd_* rose again to 0.44 ± 0.01. These results indicate that the PVA concentration affects the samples’ transmission properties, with a decrease in transmission observed at higher concentrations. Furthermore, the *T_c_* component of these samples is negligible.

The data analysis of MW2 samples shows similar trends to those observed in MW1. When the concentration of PVA increases from 7% to 15%, the *R_cd_* reaches its lowest point at 12% and then slightly increases at 15%. This implies that there is a non-linear relationship between Rcd and PVA concentration. The *T_cd_* remains stable at 0.34 ± 0.09 as the PVA concentration rises from 7% to 12%, indicating that changes in PVA concentration do not significantly impact this range. Nevertheless, at 15%, *T_cd_* decreases to 0.29 ± 0.16, suggesting that higher PVA concentrations could significantly impact this parameter. Finally, Tc generally increases as PVA concentration increases, with a slight decrease at 12% followed by a rise at 15%.

The inverse Monte Carlo method [35] was then employed to calculate the hydrogels’ $\left(\mu_{a}\right)$ and $\left(\mu_{s}\right)$ coefficients. This method compared the measured values of reflection (*R_cd_)* and transmission (*T_cd_* and *T_c_*) coefficients with the ones generated by proposing a set of optical properties of the medium. The optimization was guided by the downhill simplex algorithm, emphasizing the importance of suitable initial parameter values for convergence.

### 4.5. Porosity Distribution

**Figure 10:**
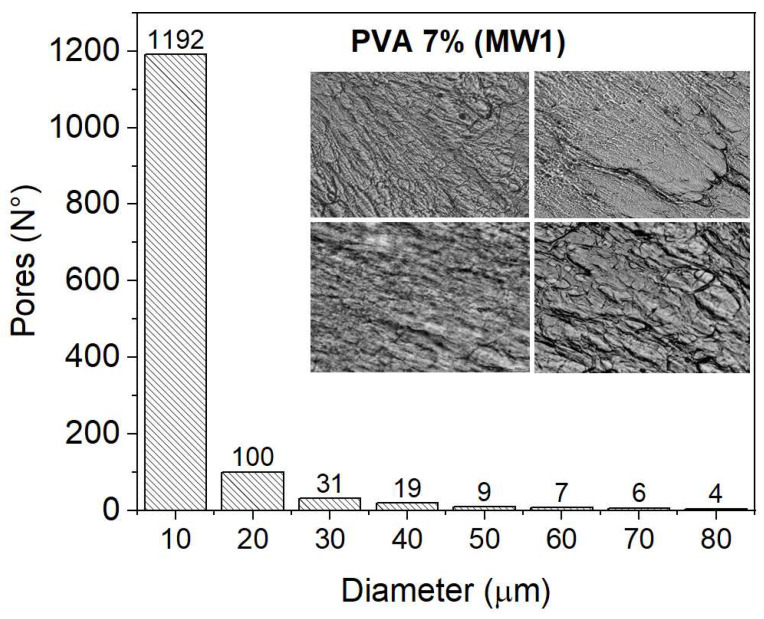
Histogram of the number of pores versus the diameter of the samples (measuring 1 cm × 1 cm × 50 µm) taken from a section of the wall of the PVA MW1: 7% hydrogel. Each measurement is an average of four samples.

The number and diameter of pores were determined using microscopic images of PVA hydrogels (Nikon optical microscope, Tokyo, Japan 40X objective, 10X eyepiece). The images were processed using ImageJ and MATLAB programs, which utilized binarization and filters. The samples measured 1 cm × 1 cm × 50 µm and were obtained from sections of one wall of each hydrogel using a Leica Biosystems cryostat. As the concentration of PVA increases, the number of pores decreases due to a reduction in diameter. Figure 10 shows the histogram of the number of pores versus the diameter of the PVA MW1: 7% hydrogel. Each measurement is an average of four samples.

This study analyzes the distribution of porosity in nine hydrogels (MW1: 7%, 9%, 12%, 15%, 20%, and MW2: 7%, 9%, 12%, 15%). The results section displays histograms for all hydrogels analyzed.

### 4.6. Density and Elasticity Modulus

Four samples measuring 1 cm × 1 cm × 0.2 cm were cut from one of the PVA hydrogel walls to determine the density and elasticity modulus (Young’s modulus). The density was measured using the equation ρ=m/V according to Archimede’s principle and the elasticity modulus of Young’s modulus (E=σ/ε) was determined using a traction device developed by the Research and Technological Development Unit of the General Hospital of Mexico “Dr. Eduardo Liceaga”.

## Figures and Tables

**Table 1:** Physical and optoacoustic variables of the photoacoustic signals of all the study hydrogels. The values are the average of three measurements plus their standard deviation.

MW1				
PVA (%)	*D* (mm)	$t_{a}(\mu s)$	*c* (m/s)	*A* (mV)
7	39.70 ± 1.34	25.60 ± 0.06	1551 ± 4.79	0.87 ± 0.06
9	43.16 ± 0.31	28.18 ± 0.02	1532 ± 0.60	1.51 ± 0.02
12	45.00 ± 0.24	28.45 ± 0.03	1582 ± 3.32	1.70 ± 0.14
15	46.22 ± 0.48	30.14 ± 0.06	1534 ± 6.38	4.07 ± 2.76
20	46.62 ± 0.26	30.82 ± 0.07	1513 ± 0.67	6.66 ± 0.41
**MW2**				
7	40.20 ± 0.65	26.39 ± 0.06	1523 ± 2.62	1.50 ± 0.31
9	40.54 ± 2.00	27.08 ± 0.01	1497 ± 1.50	1.59 ± 0.17
12	43.84 ± 0.59	28.63 ± 0.04	1531 ± 0.15	3.41 ± 0.12
15	43.89 ± 0.22	28.67 ± 0.06	1531 ± 1.16	4.06 ± 0.63

**Table 2:** Sample’s optical properties as a function of the PVA concentration.

	MW1			
PVA (%)	*µ_a_* (cm^−1^)	*µ_s_* (cm^−1^)	*µ_s_’* (cm^−1^)	*g*
7	0.05 ± 0.00	54.94 ± 0.55	20.60 ± 0.21	0.62 ± 0.01
9	0.06 ± 0.00	55.30 ± 0.55	21.01 ± 0.21	0.62 ± 0.01
12	2.17 ± 0.00	51.82 ± 0.10	10.36 ± 0.02	0.80 ± 0.00
15	2.78 ± 0.02	52.57 ± 0.42	9.72 ± 0.08	0.82 ± 0.01
20	0.58 ± 0.01	53.15 ± 0.42	17.54 ± 0.14	0.67 ± 0.01
	**MW2**			
7	0.23 ± 0.01	12.86 ± 0.64	9.64 ± 0.48	0.25 ± 0.01
9	0.19 ± 0.02	9.88 ± 1.17	6.91 ± 0.83	0.30 ± 0.04
12	0.18 ± 0.02	11.01 ± 1.10	7.70 ± 0.77	0.30 ± 0.03
15	0.20 ± 0.02	10.35 ± 1.04	8.07 ± 0.81	0.22 ± 0.02

**Table 3:** Dimensions of the PVA hydrogels for two MWs and different concentrations. The measurements are the average plus the standard deviation of three hydrogels.

PVA: MW1					
Diameter (mm)	7%	9%	12%	15%	20%
d_1_	8.39 ± 0.07	9.82 ± 0.04	10.42 ± 0.18	10.88 ± 0.12	11.20 ± 0.52
d_2_	22.60 ± 0.82	23.86 ± 0.14	24.44 ± 0.24	24.62 ± 0.23	24.32 ± 0.20
d_3_	8.68 ± 0.52	9.47 ± 0.22	10.14 ± 0.30	10.72 ± 0.12	11.09 ± 0.46
D	39.70 ± 1.34	43.16 ± 0.31	45.00 ± 0.24	46.22 ± 0.48	46.62 ± 0.26
**PVA: MW2**					
d_1_	8.88 ± 0.28	9.30 ± 1.12	10.62 ± 0.08	11.00 ± 0.22	-
d_2_	21.88 ± 0.06	22.00 ± 1.77	23.32 ± 0.04	23.92 ± 0.26	-
d_3_	9.01 ± 0.41	9.30 ± 0.26	9.56 ± 0.48	9.66 ± 0.04	-
D	40.20 ± 0.65	40.54 ± 2.00	43.84 ± 0.59	43.89 ± 0.22	-

**Table 4:** Sample’s optical properties as a function of the PVA concentration with a width of 100 µm.

	PVA: MW1			PVA: MW2		
PVA (%)	*R_cd_*	*T_cd_*	*T_c_*	*R_cd_*	*T_cd_*	*T_c_*
7%	0.44 ± 0.01	0.47 ± 0.01	0.0	0.23 ± 0.05	0.34 ± 0.09	0.23 ± 0.08
9%	0.44 ± 0.01	0.44 ± 0.01	0.0	0.19 ± 0.12	0.34 ± 0.09	0.33 ± 0.12
12%	0.17 ± 0.00	0.42 ± 0.05	0.0	0.18 ± 0.10	0.34 ± 0.05	028 ± 0.06
15%	0.14 ± 0.01	0.41 ± 0.01	0.0	0.20 ± 0.10	0.29 ± 0.16	0.31 ± 0.08
20%	0.34 ± 0.01	0.44 ± 0.01	0.0	-	-	-

## Data Availability

Not applicable.
