# Correction: Ramírez-Chavarría et al. Study of Polyvinyl Alcohol Hydrogels Applying Physical-Mechanical Methods and Dynamic Models of Photoacoustic Signals. *Gels* 2023, *9*, 727

**DOI:** 10.3390/gels11050360

**Published:** 2025-05-14

**Authors:** Roberto G. Ramírez-Chavarría, Argelia Pérez-Pacheco, Emiliano Terán, Rosa M. Quispe-Siccha

**Affiliations:** 1 Engineering Institute, National Autonomous University of Mexico, Mexico City 04510, Mexico; rramirezc@iingen.unam.mx; 2 Research and Technological Development Unit, Research Department, General Hospital of Mexico “Dr. Eduardo Liceaga”, Mexico City 06726, Mexico; argeliapp@ciencias.unam.mx; 3 Faculty of Physical-Mathematical Sciences, Autonomous University of Sinaloa, Culiacan, Sinaloa 80040, Mexico; eteran@uas.edu.mx

## Text Correction

In the original publication [1], there was a mistake in Equation (8) as published. There was a mistake in the unit and the sign in Equation (8). The corrected Equation (8) appears below.

(8) $$W_{PVA}\left(g\right)=\left[PVA\;concentration\;\left(\%\right)\times H_{2}O\;volume\;(mL)\right]/100\%$$

The authors state that the scientific conclusions are unaffected. This correction was approved by the Academic Editor. The original publication has also been updated.
